# Pathologic N_0_ Status in Clinical T_1_N_0_M_0_ Lung Adenocarcinoma is Predictable by the Solid Component Proportion with Quantitative CT Number Analysis

**DOI:** 10.1038/s41598-017-16701-x

**Published:** 2017-12-01

**Authors:** Meng Li, Ning Wu, Li Zhang, Wei Sun, Jianwei Wang, Lv Lv, Jiansong Ren, Dongmei Lin

**Affiliations:** 1 Department of Diagnostic Radiology, National Cancer Center/Cancer Hospital, Chinese Academy of Medical Sciences and Peking Union Medical College, Beijing, 100021 China; 2 PET-CT center, National Cancer Center/Cancer Hospital, Chinese Academy of Medical Sciences and Peking Union Medical College, Beijing, 100021 China; 3 Department of Pathology, Beijing Cancer Hospital, Beijing, 100142 China; 4 National Office for Cancer Prevention and Control, National Cancer Center/Cancer Hospital, Chinese Academy of Medical Sciences and Peking Union Medical College, Beijing, 100021 China

## Abstract

Correctly predicting pathologic regional node-negative (pN_0_) disease in patients with lung cancer before operation may avoid unnecessary mediastinal lymph node dissection (MLND). In this study, we analyze the value of the radiographic and histopathological features of primary tumors for predicting pN_0_ status in cT_1_N_0_M_0_ lung adenocarcinoma and to establish an optimal surgical strategy for avoiding MLND in cT_1_N_0_M_0_ lung adenocarcinoma patients. We retrospectively investigated the histopathological and radiographic data of 348 surgically resected cT_1_N_0_M_0_ lung adenocarcinoma patients with systematic lymph node dissection from January 2005 to December 2012. Histopathological features and radiographic features were analyzed. Multivariable analysis was used to identify significant predictors of pN_0_ disease. Our results showed that pN_0_ disease was detected in 306 patients (87.9%) among the 348 patients with cT_1_N_0_M_0_ lung adenocarcinoma. A decreasing trend of the pN_0_ disease proportion was observed with both increasing histological grade and decreased differentiation (*P* < 0.001). In multivariable analysis, the solid component proportion was a significant predictor of pN_0_ disease. Among 110 patients with a solid component proportion of no more than 21.3%, mediastinal lymph node involvement was not observed. Patients who meet this criterion may be successfully managed with lung resection without MLND.

## Introduction

Anatomical pulmonary resection in combination with mediastinal lymph node dissection (MLND) or sampling remains the standard surgical approach even for early-stage non–small-cell lung cancer (NSCLC) patients^1,2^. However, the nodal metastatic rate among early NSCLC patients is not high. The reported nodal metastatic rate is 16% to 29% in early-stage NSCLC patients with a peripheral tumor 3 cm or less in diameter, 15% to 20% in patients with a peripheral tumor 2 cm or less^3–5^, and as low as 6.6% in clinical T_1a_N_0_M_0_ lung adenocarcinoma patients^6^. Considering the surgical injury, MLND is unnecessary and harmful to patients without mediastinal node involvement^2,7,8^. Correctly predicting pathologic node-negative (pN_0_) disease in patients with NSCLC before operation may avoid unnecessary MLND, although it is an extremely difficult problem in clinical practice. Because the relative low diagnostic sensitivity, these tumors with no nodal involvement on computed tomography (CT) or even positron emission tomography (PET)/CT image, which are staged as clinical N_0_ (cN_0_) disease, may also have pathological regional lymph node metastases^9,10^. Consequently, MLND is still adopted as a gold standard in almost NSCLC surgical treatments.

Recent advancements in the quality of high-resolution CT (HRCT) and the widespread practice of low-dose CT (LDCT) for lung cancer screening have resulted in a greater increase in the early detection of smaller-sized NSCLC, especially lung adenocarcinoma appearing as subsolid nodule^11,12^. As a result, limited resection like wedge resection or segmenectomy is more favored and the necessity of standard lobectomy begin to be questioned for small or subsolid types of adenocarcinoma^2^. Meanwhile, the degree to which MLND is necessary also becomes a challenging issue because the biological behavior of small lung adenocarcinomas showing as subsolid nodules on CT is less aggressive and usually pN_0_ diseases. These patients are good candidates for the omission of MLND. Therefore, it is very important to establish reliable prediction criteria for pN_0_ disease preoperatively especially using quantitative radiographic imaging to avoid performing MLND.

In this study, we attempt to analyze the value of radiographic and histopathological features of primary tumors for predicting pN_0_ disease in cT_1_N_0_M_0_ lung adenocarcinoma to establish an optimal surgical strategy for avoiding unnecessary MLND in these patients. We analyzed both HRCT and PET/CT indexes, and we also proposed three-dimensional computer quantified CT number analysis to calculate the solid component (Sc) proportion and ground glass opacity component (GGOc) proportion.

## Results

### Patient demographics

The clinicopathological characteristics of the 348 patients with lung adenocarcinoma included in this study are summarized in Table 1. Of the 348 patients, 306 (87.9%) had pN_0_ disease, and 42 (12.1%) had nodal metastasis. The pN_0_ disease proportion was 83.6% (122/146) in males and 91.1% (184/202) in females (*P* = 0.033).

**Table 1:** Patient characteristics.

Characteristics	No. of patient (%)
Median age (y)^a^	58.4 ± 9.8 (31~84)
Sex	
Male	146 (42.0)
Female	202 (58.0)
Smoking status	
Current or former smoker	109 (31.3)
Nonsmoker	239 (58.0)
Invasive lobe	
RUL	139 (39.9)
RML	19 (5.5)
RLL	62 (17.8)
LUL	80 (23.0)
LLL	48 (13.8)
Surgical procedure	
Wedge resection	25 (7.2)
Segmentectomy	1 (0.3)
Lobectomy	322 (92.5)
Imaging technique^b^	
CT	348 (100)
PET/CT	137 (39.4)
Subtype prodominance	
AIS	36 (10.3)
MIA	44 (12.6)
Lepidic	43 (12.4)
Acinar	170 (48.9)
Papillary	30 (8.6)
Micropapillary	5 (1.4)
Solide	10 (2.9)
Variants	10 (2.9)
N Stage	
N_0_	306(87.9)
N_1_	21(6.0)
N_2_	21(6.0)
Pathologic Stage	
IA	164 (47.1)
IB	142 (40.8)
IIA	21 (6.0)
IIIA	21 (6.0)

Note. –Unless otherwise indicated, data are numbers, with percentages in parentheses. ^a^Data are means ± standard deviations, with range in parentheses. ^b^All patients underwent CT examination. AIS, adenocarcinoma *in situ*; MIA, minimally invasive adenocarcinoma; LLL, left lower lobe; LUL, left upper lobe; RLL, right lower lobe; RML, right middle lobe; RUL, right upper lobe.

### Correlation between pN_0_ disease proportion and pathological features

The pN_0_ disease proportion in histological Grade 1, Grade 2, Grade 3 and Grade 4 was 100%, 97.7%, 82.5% and 73.3%, respectively (Table 2). The pN_0_ disease proportion in the well-differentiated group, moderately differentiated group and poorly differentiated group was 97.7%, 80.6% and 65.5%, respectively (Table 3). Decreasing trends were observed for both histological grade and differentiation grade (*P* < 0.001).

**Table 2:** Trend of pN_0_ disease proportion among different tumor grades.

Tumor grades	pN_0_ disease proportion	*P*
Grade 1	80/80 (100%)	<0.001
Grade 2	42/43 (97.7%)	
Grade 3	165/200 (82.5%)	
Grade 4	11/15 (73.3%)	
Total	298/338 (88.4%)	

Note. – 10 patients with invasive adenocarcinoma of a variant pattern were not included; pN_0_, pathologic node-negative.

**Table 3:** Trend of pN_0_ disease proportion among different tumor differentiation degrees.

Tumor differentiation degrees	pN_0_ disease proportion	*P*
Well-differentiated group	171/175 (97.7%)	<0.001
Moderately differentiated group	116/144 (80.6%)	
Poorly differentiated group	19/29 (65.5%)	
Total	306/348 (87.9%)	

Note. – pN_0_, pathologic node-negative.

### Radiographic features analysis

**Figure 1:**
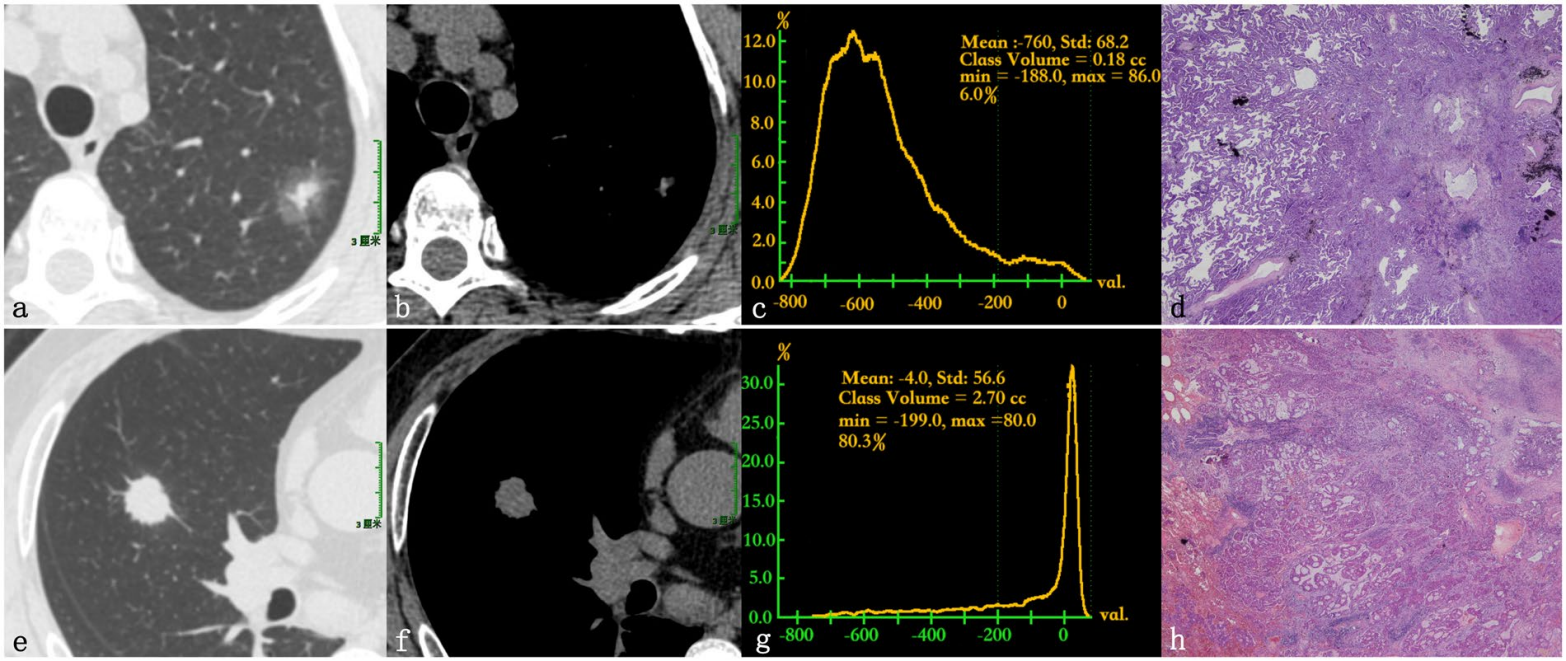
Images of a 59-year-old female patient with invasive adenocarcinoma (**a**–**d**) and a 45-year-old male patient with invasive adenocarcinoma (**e**–**h**). (**a**,**b**) The lung window and mediastinal window of the axial HRCT showed a partially solid nodule in the left upper lobe. (**c**) The CT number distribution curve revealed that the solid component proportion of the nodule was 6%. (**d**) The photomicrograph (hematoxylin and eosin staining; magnification × 20) revealed a lepidic-predominant invasive adenocarcinoma consisting of 80% lepidic pattern and 20% acinar pattern (pT_1_N_0_M_0_). (**e**,**f**) The lung window and mediastinal window of the axial HRCT showed a solid nodule in the right upper lobe. (**g**) The CT number distribution curve revealed that the solid component proportion of the nodule was 80.3%. (**h**) The photomicrograph (hematoxylin and eosin staining; magnification × 100) revealed an acinar-predominant invasive adenocarcinoma consisting of 80% acinar pattern and 20% solid pattern (pT_1_N_2_M_0_, right superior mediastinal lymph node metastasis, 3/6; subcarinal lymph node metastasis, 1/1).

**Figure 2:**
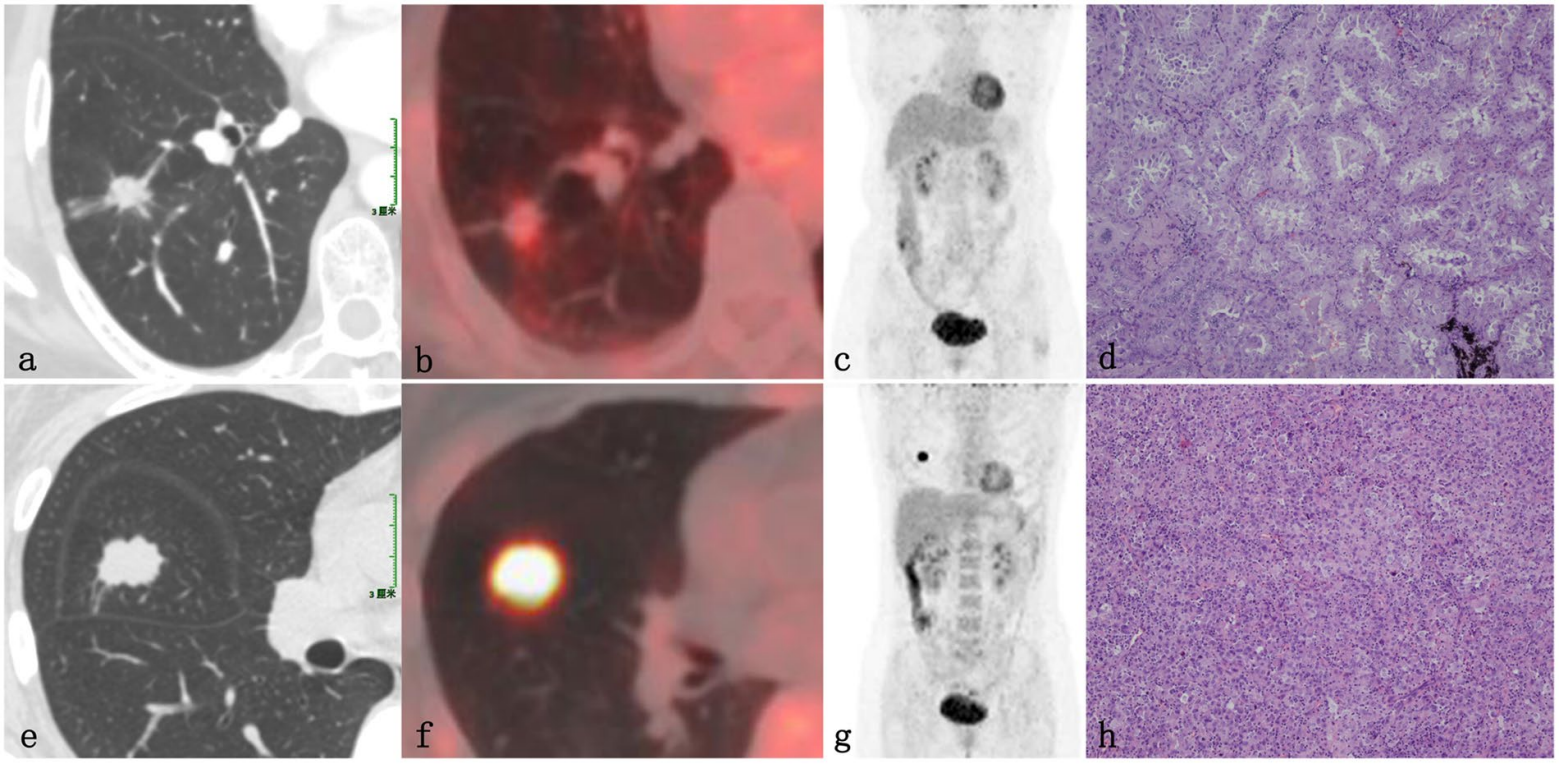
Images of a 59-year-old female patient with invasive adenocarcinoma (**a–d**) and a 46-year-old female patient with invasive adenocarcinoma (**e–h**). (**a**) The lung window of the axial HRCT showed a solid nodule in the right lower lobe. (**b**) PET/CT revealed low metabolic activity (SUV_max_, 1.5). (**c**) PET showed no other abnormal FDG uptake in the whole body. (**d**) The photomicrograph (hematoxylin and eosin staining; magnification × 100) indicated an acinar predominant invasive adenocarcinoma consisting of 60% acinar pattern, 35% lepidic pattern and 5% papillary pattern (pT_1_N_0_M_0_). (**e**) The lung window of the axial HRCT showed a solid nodule in the right lower lobe; the nodule had a lobulated and speculated margin. (**f**) PET/CT revealed high metabolic activity (SUV_max_, 7.8). (**g**) PET showed no other abnormal FDG uptake in the whole body. (**h**) The photomicrograph (hematoxylin and eosin staining; magnification × 100) indicated a solid predominant invasive adenocarcinoma consisting of 70% solid pattern and 30% acinar pattern (pT_1_N_1_M_0_, parabronchial lymph node metastasis of the right middle lobe, 1/3).

The univariate analysis of radiographic features of node-negative and node-positive disease is presented in Table 4. Tumor size, size stratification, appearance, location, contour, GGOc proportion, Sc proportion and maximum SUV (SUV_max_) differed significantly between pN_0_ disease and node-positive disease (*P* = 0.002, *P* = 0.008, *P* < 0.001, *P* = 0.032, *P* = 0.006, *P* < 0.001, *P* < 0.001, *P* = 0.001, respectively). Specific to quantitative radiographic indexes, the pN_0_ disease had more GGOc proportion, less Sc proportion (Fig. 1) and lower SUV_max_ (Fig. 2).

**Table 4:** Correlation between radiographic features and lymph node status.

Radiographic features	Node-positive	Node-negative	χ^2^/*t*	*P*
**HRCT**	42	306		
Size^**a**^	2.20 ± 0.51	1.90 ± 0.58	−3.143	0.002
Size stratification			9.556	0.008
≤1 cm	0	21		
1–2 cm	17	173		
>2 cm	25	112		
Appearance				
GGO	0	52	43.980	<0.001
PSN	4	142		
SN	38	112		
Location			4.579	0.032
Center	12	47		
Periphery	30	259		
Contour			10.127	0.006
Smooth	2	67		
Lobular	20	156		
Spiculated	20	86		
Necrosis			1.482	0.223
Yes	6	26		
No	36	280		
**Quantified CT number analysis**				
GGOc proportion^**a**^	(5 ± 6) %	(24 ± 25) %	11.522	<0.001
Sc proportion^**a**^	(82 ± 17) %	(42 ± 32) %	−12.799	<0.001
**PET/CT**	137	10		
SUV_max_	5.23 ± 2.71	3.20 ± 2.77	−3.510	0.001

Note. –Unless otherwise indicated, data are number of patients. ^a^Data are means ± standard deviation. HRCT, high-resolution computer tomography; GGOc, glass-ground-opacity component; Sc, solid component; PET, positron emission tomography; SUV, standard uptake value.

**Figure 3:**
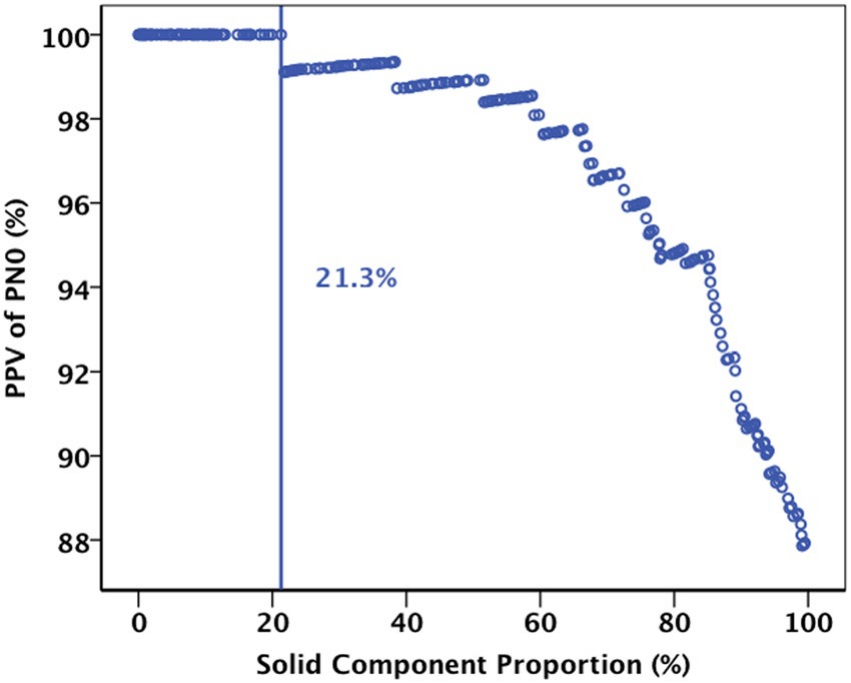
The Positive predictive value (PPV) of pN_0_ disease in cT_1_N_0_M_0_ lung carcinoma based on the tumor solid component (Sc) proportion.

Logistic regression analysis demonstrated that the Sc proportion was only significant predictor of pN_0_ disease (β = 6.153, *P* = 0.001; *OR* = 470.085, 95% *CI*: 12.291~17978.987). With the increase of Sc proportion, the chance of pN_0_ disease is reduced (Fig. 3). The positive predictive value (PPV) of pN_0_ disease was 100% for patients with a tumor Sc proportion of no more than 21.3%. Of the 348 patients, only 137 underwent PET/CT, and thus SUV_max_ was removed from the multivariate analysis.

## Discussion

A randomized, prospective clinical trial has suggested that MLND can be avoided if there is sufficient evidence of pN_0_ disease^13^. Although multiple studies have attempted to identify clinical, pathological and radiographic factors of lymph node status in early-stage adenocarcinoma^6,7,14–18^, our study differs from these previous studies in several ways. First, this study made cT_1_N_0_M_0_ lung adenocarcinoma a separate object of study. Second, all resected specimens were reviewed pathologically according to the 2011 IASLC/ATS/ERS classification, and a tumor-grading system was established based on histopathological subtypes according to the newly proposed classification^19^. Third and most important, quantitative radiographic analysis method was introduced, the Sc and GGOc proportion calculated using three-dimensional computer-quantified CT number analysis was adopted to qualitatively reflect the tumor internal appearance, resulting in more objective and convenient selection criteria for clinical application.

Our study results included three main findings: (a) the Sc proportion was an independent predictor of pN_0_ disease in cT_1_N_0_M_0_ lung adenocarcinoma; (b) all cT_1_N_0_M_0_ lung adenocarcinoma patients with an Sc proportion of no more than 21.3% had no lymph node metastasis; and (c) the pN_0_ disease proportion exhibited a decreasing trend with both increasing histological grade and differentiation grade. Our results may have clinical implications. With the aid of preoperative quantitative radiographic features, we differentiated cT_1_N_0_M_0_ lung adenocarcinoma patients with or without pN_0_ disease, thus reducing unnecessary MLND.

In our study, tumors were graded according to four classifications based on histopathological subtypes and prognosis. Sica *et al*.^20^ first proposed a 3-tier grading system for lung adenocarcinoma in 2010: Grade 1, a pattern with low metastatic potential (bronchioloalveolar carcinoma, BAC); Grade 2, patterns with intermediate metastatic potential (acinar and papillary); and Grade 3, patterns with high metastatic potential (solid and micropapillary). However, in 2011, the newly proposed lung adenocarcinoma classification discontinued the use of “BAC”, and other changes in uniform terminology, such as adenocarcinoma *in situ* (AIS) and minimally invasive adenocarcinoma (MIA), and diagnostic criteria were implemented^19^. Studies^21,22^ have shown that this new classification system is a stage-independent predictor of survival. Five-year disease-free survival is 100% or near 100% in patients with AIS and MIA, more than 85% in lepidic predominant invasive adenocarcinoma, 65% in acinar and papillary predominant invasive adenocarcinoma, and 40% in solid and micropapillary predominant invasive adenocarcinoma. The tumor-grading system in this study is based on a combination of the classification of Sica *et al*. and the lung adenocarcinoma classification system proposed in 2011. A higher grade represents a worse prognosis.

A decreasing trend in the pN_0_ disease proportion with increasing tumor grade and decreasing tumor differentiation was observed in this study, consistent with the worse prognosis of lung adenocarcinoma patients with higher tumor grade and poorer tumor differentiation. In this study, all cT_1_N_0_M_0_ lung adenocarcinoma patients with grade 1 tumors (AIS or MIA) had pN_0_ disease, whereas only 97.7% of patients with well-differentiated tumors had pN_0_ disease. Therefore, we believe that tumor grade has stronger predictive ability for lymph node status than tumor differentiation and that tumor grade 1 is a significant predictor of pN_0_ disease. However, because of histological heterogeneity, AIS and MIA can only be diagnosed in resected specimens and not in small biopsies or cytology specimens^23^. Consequently, histopathological subtypes have limited significance in predicting node metastasis preoperatively and determining the most appropriate surgical procedures.

In univariate analysis of radiographic features, tumor size, size stratification, location, contour, appearance, Sc proportion, GGOc proportion and SUV_max_ were associated with pN_0_ disease. In this study, all 21 patients with a tumor size of no more than 1 cm had pN_0_ disease, in accordance with previous study^14^. We also found that compared with patients with node-positive disease, primary tumors in patients with pN_0_ disease are more likely to present a smooth margin and peripheral location. Smooth margin and peripheral location were frequently proven to be benign signs in lung nodule diagnosis. Tumor appearance has been proven to be a significant predictor of lymph node status. Researchers have found that most patients with a dominant GGO component on CT have no lymph node metastasis^15^. In this study, all 52 patients with pGGO had pN_0_ disease, consistent with previous studies^6,16^. However, tumor appearance is only used as a subjective and visual characteristic in the past study, and thus is not sufficiently objective and accurate for prediction of pN_0_ disease. The great progresses of computer make quantitative imaging increasingly applying in modern radiology practice, assisting in the clinical assessment and providing a source of biomarkers for a spectrum of diseases^24^. In the current study, the Sc proportion and GGOc proportion calculated by three-dimensional computer-quantified CT number analysis were adopted to qualitatively reflect tumor appearance innovatively, resulting in more objective and accurate selection criteria for pN_0_ disease. The Sc proportion and GGOc proportion were all associated with lymph node status. Primary tumors in patients with pN_0_ disease had lower Sc proportions and higher GGOc proportions than patients with node-positive disease. Studies^7,17,18^ have demonstrated that the solid component is a powerful predictor of lymph node metastasis, consistent with our study. SUV_max_ is a semiquantitative index of FDG uptake of PET/CT, which is associated with glucose metabolism in tissue. SUV_max_ was significantly lower in patients with pN_0_ disease than patients with node-positive disease in this study, consistent with previous studies^7,25–27^.

We analyzed all factors except SUV_max_ by logistic multivariate analysis to assess the joint effects and interactions of the variables on pN_0_ disease. Our results showed that only the Sc proportion retained statistical significance, whereas tumor size, size stratification, location, contour, appearance and GGOc proportion did not differ significantly in multivariate analysis. We believe that the effects of these factors were substituted by the enrolled index. When Sc proportion ≤21.3% was used as the criterion, the PPV for pN_0_ disease was 100%. On the basis of our result, patients of cT_1_N_0_M_0_ lung adenocarcinoma who meet this reliable prediction criterion may omit MLND when surgical treatment.

Our study has several limitations. First, this is a single study; further studies at multiple centers are needed to confirm our results, especially the predictive value of Sc. Second, this is a retrospective study, and a prospective study is needed to verify this conclusion. Finally, we did not perform survival analysis because the follow-up time was too short in some cases; advanced study is warranted to analyze patient survival.

In conclusion, we respectively reviewed 348 cT_1_N_0_M_0_ lung adenocarcinoma patients who underwent surgical pulmonary resection with systematic MLND at our hospital in an attempt to investigate independent predictors of pN_0_ disease. The Sc proportion was identified as statistically significant predictive risk factor. The PPV of pN_0_ disease was 100% for patients with a tumor Sc proportion of no more than 21.3%. Patients who meet this criterion may be managed with lung resection without MLND.

## Materials and Methods

### Patients

This prospective study was approved by the institutional review board at the Cancer Hospital of the Chinese Academy of Medical Sciences, and the informed consent was waived due to the retrospective nature of the study and the data were going to be analyzed anonymously. This study was conducted in accordance with the Declaration of Helsinki.

A total of 348 consecutive patients with surgically resected cT_1_N_0_M_0_ lung adenocarcinoma at our hospital between January 2005 and December 2012 were included. The inclusion criteria for our study were as follows: **(a)** single adenocarcinomas (3 cm or less in diameter at HRCT) with no evidence of malignant satellite nodules (as proven by prior imaging study or lung biopsy); **(b)** no hilar or mediastinal lymphadenopathy on imaging study or at mediastinoscopy; a short lymph node diameter greater than 1 cm on CT axial image with window level in 380 HU and window within 40 HU or maximum SUV (SUV_max_) on PET/CT image more than 2.5 was determined as lymphadenopathy; **(c)** first treatment with surgical pulmonary resection plus systematic MLND (more than 6 hilar or mediastinal lymph nodes resected); **(d)** either integrated ^18^F fluorodeoxyglucose (^18^F-FDG) PET/CT or chest HRCT studies acquired within 1 month before resection; **(e)** both pathological sections and clinical data were available for review.

### Imaging and Interpretation

All 348 patients included in this study had undergone chest HRCT for preoperative staging. HRCT images were obtained with an eight- (LightSpeed Ultra, GE Medical Systems), 16- (ProSpeed or Discovery ST, GE Medical Systems) or 64- (LightSpeed VCT, GE Medical Systems or Toshiba Aqulion, TOSHIBA Medical Systems) slice spiral CT scanner. Unenhanced CT images were obtained at 120 kVp and 250~350 mA with a reconstruction kernel with a standard algorithm. The reconstruction thickness was 1.0 or 1.25 mm, and the interval was 0.8 or 1.0 mm. A total of 182 patients underwent enhanced HRCT examination, in which 60 to 80 ml of intravenous contrast was administered at 2.0 to 2.5 mL/s and enhanced images were obtained 25 to 30 seconds after contrast infusion.

HRCT images were assessed retrospectively for morphological features and three-dimensional computer-quantified CT number analysis. Two radiologists (M.L. and L.Z., 11 and 8 years of experience, respectively), who were informed that the involved patients had surgically treated adenocarcinoma but were blinded to histological subtypes, analyzed the morphological features on Carestream GCRIS 2.1 PACS workstation (Carestream Health) in consensus. The morphological features included tumor size (the longest tumor diameter on the transverse lung window image in which the largest nodule dimension appeared), appearance (solid nodule [SN], part-solid nodule [PSN], or pure ground-glass-opacity [pGGO])^28^, location (center or periphery, periphery was defined as within 3 cm of the pleura)^29^, and contour (smooth, lobular or spiculated), necrosis (necrosis of tumor was defined as low attenuation in the tumor). Three-dimensional computer-quantified CT number analysis was performed on an ADW 4.6 workstation (GE Medical Systems) to measure the proportion of GGOc and Sc. This measurement was performed in two steps. For the first step, the entire tumor mass was separated from surrounding anatomic structures using computer-aided volume measurement software (Auto Contour in Volume Rendering, GE Medical Systems). The computer generated tumor boundaries were then visually inspected by a radiologist (L.Z.) for accuracy and consistency. If any segmentation results were considered suboptimal, the tumor contours that were superimposed on the original images were edited by the same radiologist (L.Z.), and then the mean CT value of tumor was automatically calculated by computer. Threshold values of GGOc and Sc were obtained by receiver operating characteristic (ROC) curves. As the second step, the entire tumor mass was separated using the same method in the first step, and the computer automatically generated the CT number histogram of the tumor. The proportion of GGOc/Sc was calculated based on the threshold values obtained in the first step. The calculation methods were also described in detail in our previous report^30^.

Two chest radiologists with PET/CT diagnostic experience prospectively evaluated the integrated PET/CT images. For semiquantitative analysis of ^18^F-FDG uptakes, a region of interest (ROI) was placed over the tumor site on the hottest trans-axial slice. In some patients, nodular FDG uptake could not be identified on the PET component images of the PET/CT study. In those patients, a ROI was drawn in a presumed nodular location, taking into account the CT component images of PET/CT. The SUV_max_ within the ROI was used as the reference measurement.

### Pathologic Evaluation

All resected specimens were reviewed pathologically by an experienced lung pathologist (S.W., 8 years of experience in lung pathology) according to the 2011 International association for the study of lung cancer/American thoracic society/European respiratory society (IASLC/ATS/ERS) classification^19^. For difficult cases, histological subtypes were assessed by consultation with pathological experts. Based on the reported prognosis of lung adenocarcinoma^31–34^, tumors were graded according to four classifications. Grade 1 included histological subtypes of adenocarcinoma *in situ* (AIS) and minimally invasive adenocarcinoma (MIA). Grade 2 corresponded to the lepidic pattern of invasive adenocarcinoma. Grade 3 corresponded to tumors that showed acinar or papillary patterns. Grade 4 corresponded to tumors that showed micropapillary or solid patterns. When tumors were composed predominantly of a variant pattern, the tumors were removed from this classification system because they were uncommon and heterogeneous in terms of biological behavior and prognosis. Considering the proximity of tumor cells and normal cells under the microscope, tumors were divided into the well-differentiated group, moderately differentiated group and poorly differentiated group.

### Statistical analysis

The trends of pN_0_ disease proportion among the different tumor grades and tumor differentiation degrees were assessed by chi-square trend test. The prevalences of nominal variables (e.g., tumor appearance, location, contour and intratumoral necrosis) were compared using Fisher’s exact test. Differences in the mean values of continuous variables (e.g., tumor size, GGOc proportion, Sc proportion and SUV_max_) were compared using the Independent-Student’s *t* test. Multivariate logistic regression analysis was used to identify significant predictors of pathologic node-negative disease. All statistical analyses were performed using a commercial software package (SPSS, Inc., an IBM Company, Chicago, IL, USA). A *P* value of less than 0.05 was considered to indicate a significant difference.

### Data availability

All data generated or analysed during this study are included in this published article (and its Supplementary Information files).

## Electronic supplementary material

Dataset 1
